# Histomorphometric bone assessment in patients with fracture of the proximal end of the femur

**DOI:** 10.1590/1413-78522015230201056

**Published:** 2015

**Authors:** Caio Gonçalves de Souza, Márcio Passini Gonçalves de Souza, Vanda Jorgetti, Luciene Machado dos Reis

**Affiliations:** 1 Universidade de São Paulo, Faculdade de Medicina, Department of Orthopedics and Traumatology, São Paulo, SP, Brazil, 1. Department of Orthopedics and Traumatology, Faculdade de Medicina da Universidade de São Paulo, São Paulo, SP, Brazil; 2 Universidade de São Paulo, Faculdade de Medicina, Laboratory of Medical Research, 2. Laboratory of Medical Research (LIM 16), Faculdade de Medicina da Universidade de São Paulo, São Paulo, SP, Brasil

**Keywords:** Bone and bones, Osteoporosis, Hip fractures

## Abstract

**OBJECTIVE:**

To determine whether there is a difference on the bone architecture in patients with femoral neck fracture compared to patients with intertrochanteric fractures and assess the importance of aging on bone microarchitecture in patients with proximal femoral fracture.

**METHODS:**

Biopsy of the iliac crest was made in seventeen patients between 55 and 90 years old who were admitted to the emergency room with fractures of the proximal end of the femur. After a small fragment was removed, we made a histomorphometric analysis of it.

**RESULTS:**

There was no significant difference between patients with femoral neck fracture and trochanteric fracture in structural parameters, formation and resorption. Comparing age groups we also did not find any significant change between the groups in the parameters volume and trabecular separation.

**CONCLUSION:**

There are no difference in the morphometric parameters analyzed between the different types of fracture and age is not a significant factor in the alteration of these parameters.

**Level of Evidence II, Diagnostic Studies.:**

## INTRODUCTION

Osteoporosis is the most common metabolic bone disease. It is characterized by decreased bone mass and microarchitectural deterioration, responsible for its higher fragility and, hence, increased risk of fracture.

According to US statistics, every year around one million and a half fractures are attributed to osteoporosis and of these, about 250 thousands are of the femoral neck. Studies on the subject often associate fractures of the proximal end of the femur with osteoporosis.^1^^-^ 3

Many studies have been developed relating these fractures with the radiological aspect of the femur^4^ or the measurement of bone mass,^5^ trying to correlate the type of fracture with radiological or densitometry indices.^6^

Recent studies have shown that thinning of bone trabeculae, with its consequent fragmentation, is responsible for the breakdown of bone tissue in the proximal femur.^7^^-^ 9 This means that not always a good amount of bone mass and bone strength promote good bone resilience, which explains the fact that people with normal bone mass (seen in tests such as bone densitometry) might have fractures due to low-energy trauma.

This is why bone biopsy with histomorphometric analysis is important, because besides evaluating the amount of bone mass, it provides information on bone architecture of the skeleton in study. Because it is an invasive examination, histomorphometry is not used routinely or to screen for possible osteoporosis or other disabilities that affect the bone.

The objective of this study was to determine whether there is a difference between the bone architecture of patients with femoral neck fracture as compared to patients with transtrochanteric fractures, to allow in the future, by comparing histomorphometry and other non-invasive tests (such as quantitative tomography), infer which people have a higher risk of fracture and even what type of fracture they could have, and also find out what is the real importance of the patients' age in bone changes that lead to fracture of the proximal end of the femur, searching for an age group at highest risk for this type of fracture.

## CASES AND METHODS

Seventeen patients with proximal femur fractures who were admitted to the emergency room were followed. They were admitted to surgical treatment of the fractures. It was reported that the cause of the fracture was falling to the ground after tripping over obstacles on the floor. It was not possible to get more details on the type of trauma. The study was approved by the Institutional Ethics Committee and the patients signed a Free and Informed Consent Form.

These patients were evaluated for the presence of other diseases, according to the hospital routine, and those with health issues other than osteoporosis, such as urinary tract infection, chronic renal failure, hypertension, pulmonary infection, bone tumors or other conditions that had forced the patient to a prolonged bed rest during the last year were excluded from the study.

Inclusion criteria were patients with femoral neck fractures with deviation classified by Garden as types III or IV,^10^ and stable transtrochanteric fractures, classified by Evans as type I.^11^ Only patients requiring surgery were included.

Exclusion criteria were abnormal laboratory tests and suspected bone metastasis from a carcinoma.

With the patient anesthetized to undergo the main surgery, and after conventional antiseptic measures, a transverse skin incision of approximately two centimeters was made, at a point located 2cm posterior to the anterior superior iliac spine, isolateral to the fracture and distal 2cm from it.

**Figure 1.**
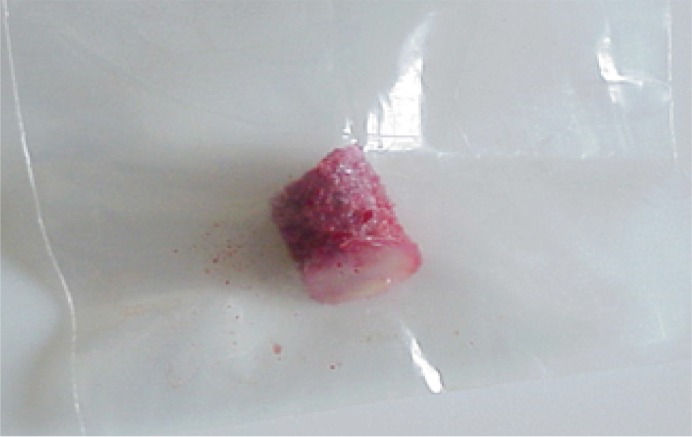
Fragment removed from the iliac crest.

After reaching the bone plane a guide was introduced, which was attached to the iliac outer cortical. Through this guide a 7mm internal diameter needle was introduced, and through exclusively clockwise rotary movements the inner cortical was perforated. The set (guide and needle) was removed by applying counter-clockwise rotational movements. The bone cylinder removed comprises, then, the trabecular bone and both cortical. (Figure 1)

After removal of the fragment and hemostasis, skin suture was made with nylon 3.0.

The fragment is placed in 70% ethanol and processed according to the following steps:^12^

• Permanence in 70% ethanol: three days
• Permanence in 100% ethanol: three days
• Permanence in toluene: one day
• Permanence in solution A (75% methyl methacrylate C_16_H_22_O_4_ + 25% dibutyl phthalate C_5_H_8_O_2_): three days
• Permanence in solution A and 1% benzoyl peroxide (Sigma C_14_H_10_O_4_): three days
• Permanence in solution A and 2.5% benzoyl peroxide: three days
• The fragment was then transferred to an incubator at 37˚C until polymerization of methyl methacrylate.
• Of each block twelve 5mm histological sections were obtained from a microtome impact (Junk K, Carl Zeiss, Germany) equipped with a tungsten knife. The cuts were, then, divided into six blades with two cuts each.
• All cuts were stained with 0.1% toluidine blue pH 6.4.

For each sample three blades were analyzed and the arithmetic mean of the values ​​obtained was made. Histological images were drawn with the aid of a cursor on a scanner board and a binocular Nikon Labophot-2A microscope, a video camera, and the software Osteomeasure, suitable for measurement of the parameters studied. This method is accurate, reproducible, it reduces the time spent in histological readings, and enables the operator to eliminate histological artifacts.^12^

The histomorphometric parameters studied follow the standardized nomenclature by the American Society of Bone and Mineral Research translated into Portuguese (except abbreviations): Trabecular volume BV/TV (%) is the volume occupied by trabecular bone, mineralized or not, expressed as percentage of the volume occupied by the bone marrow and trabeculae; Osteoid volume OV/BV (%) is the volume occupied by non-mineralized bone (osteoid), expressed as a percentage of the volume occupied by the trabecular bone (mineralized and non-mineralized); Osteoid surface OS/BS (%) is the percentage of trabecular surface covered with osteoid matrix relative to the total trabecular surface; Osteoblastic surface Ob.S/BS (%) is the percentage of trabecular surface, which presents osteoblasts relative to the total trabecular surface; Resorption surface ES/BS (%) is the percentage of the surface presenting bone resorption lacunae on the presence or absence of osteoclasts; Osteoclast surface Oc.S/BS (%) is defined the same way as osteoblast surface, applying to osteoclasts; Osteoid thickness O.Th (µm) is the thickness of the osteoid matrix edge deposited in trabecular bones, expressed in microns; Medullary volume Ma.V/TV (%): is the total percentage of bone marrow subtracted from the total trabecular volume; Trabecular thickness (or beams thickness) Tb.Th (µm) is the thickness of the trabecular bone expressed in microns; Trabecular separation (or separation of beams) Tb.Sp (µm) is the distance between the bone trabeculae expressed in microns; Trabecular number (or number of beams) Tb.N (/mm) is the number of trabecular bones, per millimeter of tissue, also being an index that expresses the trabecular density.

### Statistical analysis

The results are expressed as mean and standard deviation. The comparison between the types of fractures and gender were done using Fisher's exact test. The comparison between histomorphometric parameters was performed using the Mann-Whitney U test. The comparison by age groups was done using the Kruskal-Wallis test.

## RESULTS

We had eight males and nine females. Of these, nine had femoral neck fractures and eight had intertrochanteric fractures. The mean age was 72.9 years for patients with fracture of the femoral neck and 73.9 for patients with intertrochanteric fractures. (Table 1)

**Table 1.** Clinical characteristics of the patients.

Type of fracture	Masculine	Feminine	Age (years old)
Neck	29.4% (5)	23.5% (4)	72.9 ± 9.7
Trans	17.6% (3)	29.4% (5)	73.9 ± 9.7
Total	47.1% (8)	52.9% (9)	17 patients

Trans: Transtrochanteric

Regarding the structural parameters, there was no significant difference in the number of trabeculae, the thickness of these and trabecular separation between the two groups. (Table 2)

**Table 2.** Bone volume and structural parameters.

	Trabecular volume (%)	Nº trabeculae (Nº/mm)	Trabecular thickness (µm)	Trabecular separation (µm)
Neck	17.3 ± 5.4	1.9 ± 1.2	110.6 ± 54.0	535.9 ± 218.0
Trans	14.2 ± 4.2	1.9 ± 1.0	97.5 ± 56.0	628.3 ± 376.9

Trans: Transtrochanteric

Also in the formation parameters (osteoid volume, osteoid surface and osteoblast surface), as well as in resorption parameters (resorption surface and osteoclast surface), there were no significant changes. (Tables 3 and 4)

**Table 3.** Formation parameters.

	Osteoid volume (%)	Osteoid surface (%)	Osteoblast surface (%)
Neck	4.6 ± 11.9	9.2 ± 8.9	2.0 ± 2.8
Trans	0.7 ± 1.0	10.3 ± 11.4	1.9 ± 3.0

Trans: Transtrochanteric

**Table 4.** Reabsorption parameters.

	Reabsorption surface (%)	Osteoclast surface (%)
Neck	3.2 ± 2.6	0.3 ± 0.4
Transtrochanteric	2.0 ± 1.0	0.1 ± 0.2

By analyzing two of the parameters (trabecular volume and trabecular separation) that some studies cited as plausible to change with age, we found that in our study that did not happen, and there were no significant changes between the different age groups. (Tables 5 and 6)

**Table 5.** Trabecular volume according to age groups.

Age groups (years old)	Trabecular volume (%)
60-69	12.24 ± 4.05
70-79	17.64 ± 5.88
80-89	15.37 ± 3.09

**Table 6.** Trabecular separation according to age groups.

Age groups (years old)	Trabecular separation(µm)
60-69	702.12 ± 400.94
70-79	461.30 ± 280.43
80-89	545.80 ± 248.07

## DISCUSSION

Osteoporosis is a metabolic bone disease where there is both a reduction in the normal quantity of mineralized bone as a change in bone microarchitecture, leading to increased fracture risk in certain regions, even in the absence of high-energy trauma. It affects 25% of women over 60 years old and 5% to 10% of men in the same age group in western societies.^13^

The classification currently adopted by the World Health Organization for osteoporosis is based on densitometry evaluation, which subdivides the degree of bone loss in standard deviations below the mean for young adults (peak bone mass) in the following categories: normal, osteopenia, osteoporosis and severe or established osteoporosis.

This definition based on bone mineral density has its advantages and disadvantages. Reading errors can be caused by degenerative joint changes and osteophytes. Besides, only the measurement of bone density does not clarify the etiology of bone depletion, such as osteomalacia, whose radiological appearance is similar to that of osteoporosis.

In order to understand what makes an elderly person to lower his/her bone mass it is necessary to know the natural history of the skeleton and which are the characteristics of bone loss with aging. Two are the main changes in the skeleton with aging: Changes in the amount of bone mass and specific changes in the microarchitecture of the bone marrow. The latter change occurs more often in vertebras.^8^^,^ 14 ^,^ 15

During aging, changes in cortical and cancellous bone are not similar. Women lose 35% of the cortical bone and 50% of the cancellous bone in this processo.^8^^,^ 9 ^,^ 15 ^,^ 16 We also have different proportions of cortical and cancellous bone in frequent sites of osteoporotic fractures, for example: trochanteric region: 50% cortical to 50% spinal cord; femoral neck: 75% of cortical to 25% spinal.

Thus, people lose more cortical than cancellous bone during aging, and as we have a higher percentage of medullary bone in the trochanteric region than in the femoral neck region, it is possible that the bone microarchitecture of patients with these fractures is different, which was the original hypothesis of this study.

The cortical bone is 85% of the total body bone mass, which is measured by densitometry. After 40 years old, but especially after menopause, there is a decline in mass. This bone loss is mainly responsible for osteoporotic fractures in the hip and distal Radio regions.^3^^,^ 5 ^,^ 9

The medullary bone, however, which represents only 15% of total bone mass, has its decline from age 30, earlier than cortical bone, and does not have as significant reductions after menopause.^17^ This loss is not caused only by a narrowing of the trabecular bone that form it, but mainly by the their fragmentation. One method to evaluate changes in bone microarchitecture is histomorphometry, and for being an invasive method, it has known risks, such as pain and risk of infection, and should not be used in all patients.

In this study, we verified whether there was any difference in the analyzed parameters that could show some tendency of certain people having transtrochanteric or femoral neck fractures. After having done the statistical analysis of the data obtained, we realized that there was no statistically significant difference between the types of fracture in any of the measured parameters. This leads us to believe that a patient with an advanced level of osteopenia may suffer either a trochanteric or a femoral neck fracture. Probably what defined the type of fracture was the mechanism of trauma, rather than the bone microarchitecture.

Comparing the data obtained according the age range of patients, we expected to find differences between the surveyed values, based on the literature,^12^^,^ 16 ^,^ 18 which showed that the trabecular volume decreases and the separation of the trabeculae increases with age.^4^^,^ 13 ^,^ 19

However, when we examine the data on the trabecular volume according to age groups, we found that the average volume of patients in the 60-69 year old group was 12.24%, lower than the average in the age group 70-79, which was 17.63%, and lower than the average for patients at the 80-89 age group, which was 15.35%. We also had a female patient aged 90 years old with trabecular volume of 20.96%.

This was due to the fact that we found two patients in the 70-79 age group with trabecular volumes above 20% (26.6% and 23.3%), which increased the mean in this age group, and one patient with 69 years old with 6.1% trabecular volume, which significantly lowered the average of the five patients aged 60 to 69 years.

Analyzing another parameter, trabecular separation, which should increase with age, we realized that in the 60-69 age group, it was 702.12µm, and in the groups 70-79 and 80-89 it was 461.3µm and 545.8µm, respectively. The only patient aged 55 in the group had 680.9µm, a value greater than those aged 70-79 and 80-89.

This result was caused by the fact that we had a 60 year old patient with trabecular separation equal to 1294.1 µm, the same that had low trabecular volume (6.1%). Besides, we had two 84 year old patients that showed trabecular separation of approximately 300 µm, which significantly influenced the mean in a group of four patients (aged 80-89 years).

These results lead us to conclude that the differences between the parameters measured in this work do not depend much on age, but rather on personal factors of each patient, such as the habit of exercising or not, diet, sun exposure, weight, hormonal changes and many other factors that are often cited in the literature.^8^^,^ 20 ^-^ 24 This reinforces the need for physicians to, through clinical history and laboratory test for bone mass measurement, individually assess their patients, since It is well known that people over 80 years old with no risk factors for osteoporosis may have less chance to fracture the proximal end of the femur than people aged 60 who have all the risk factors.

## CONCLUSIONS

There is no difference in the histomorphometric variables between patients who have fractured the femoral neck or femoral trochanter.

The age of the patients studied did not affect these parameters, with some older patients having better indices than some younger patients.

## References

[1] Machado MM, Fernandes PR, Zymbal V, Baptista F (2014). Human proximal femur bone adaptation to variations in hip geometry. Bone..

[2] Gnudi S, Ripamonti C, Lisi L, Fini M, Giardino R, Giavaresi G (2002). Proximal femur geometry to detect and distinguish femoral neck fractures from trochanteric fractures in postmenopausal women. Osteoporos Int..

[3] Lopez LM, Grimes DA, Schulz KF, Curtis KM, Chen M (2014). Steroidal contraceptives: effect on bone fractures in women. Cochrane Database Syst Rev..

[4] Rubinacci A, Tresoldi D, Scalco E, Villa I, Adorni F, Moro GL (2012). Comparative high-resolution pQCT analysis of femoral neck indicates different bone mass distribution in osteoporosis and osteoarthritis. Osteoporos Int..

[5] Cummings SR, Black DM, Nevitt MC, Browner WS, Cauley JA, Genant HK (1990). Appendicular bone density and age predict hip frature in women. The Study of Osteoporotic Fractures Research Group. JAMA..

[6] Faulkner KG, Cummings SR, Black D, Palermo L, Glüer CC, Genant HK (1993). Simple measurement of femoral geometry predicts hip fracture: the study of osteoporotic fractures. J Bone Miner Res..

[7] Blain H, Chavassieux P, Portero-Muzy N, Bonnel F, Canovas F, Chammas M (2008). Cortical and trabecular bone distribution in the femoral neck in osteoporosis and osteoarthritis. Bone..

[8] Riggs BL, Wahner HW, Melton LJ 3rd, Richelson LS, Judd HL, Offord KP (1986). Rates of bone loss in the appendicular and axial skeletons of women Evidence of substantial vertebral bone loss before menopause. J Clin Invest..

[9] Emaus N, Berntsen GK, Joakimsen R, Fonnebø V (2006). Longitudinal changes in forearm bone mineral density in women and men aged 45-84 years: the Tromso Study, a population-based study. Am J Epidemiol..

[10] Garden RS (1964). Stability and union in subcapital fractures of the femur. J Bone Joint Surg Br..

[11] Jensen JS (1980). Classification of trochanteric fractures. Acta Orthop Scand..

[12] Reis LM (1998). Análise histomorfométrica de biópsias ósseas de crista ilíaca em uma amostra da população normal brasileira.

[13] Milovanovic P, Djonic D, Marshall RP, Hahn M, Nikolic S, Zivkovic V (2012). Micro-structural basis for particular vulnerability of the superolateral neck trabecular bone in the postmenopausal women with hip fractures. Bone..

[14] Darby AJ, Meunier PJ (1981). Mean wall thickness and formation periods of trabecular bone packets in idiopathic osteoporosis. Calcif Tissue Int..

[15] Mazess RB (1982). On aging bone loss. Clin Orthop Relat Res..

[16] Barger-Lux MJ, Recker RR (2002). Bone microstructure in osteoporosis: transilial biopsy and histomorphometry. Top Magn Reson Imaging..

[17] Riggs BL, Wahner HW, Dunn WL, Mazess RB, Offord KP, Melton LJ 3rd (1981). Differential changes in bone mineral density of the appendicular and axial skeleton with aging: relationship to spinal osteoporosis. J Clin Invest..

[18] Rehman MT, Hoyland JA, Denton J, Freemont AJ (1994). Age related histomorphometric changes in bone in normal British men and women. J Clin Pathol..

[19] Salamanna F, Fini M, Parrilli A, Cadossi M, Aldini NN, Giavaresi G (2013). Histological, histomorphometric and microtomographic analyses of retrieval hip resurfacing arthroplasty failed at different times. BMC Musculoskelet Disord..

[20] Dennison EM, Cooper C, Cole ZA (2010). Early development and osteoporosis and bone health. J Dev Orig Health Dis..

[21] Anderson JJ, Roggenkamp KJ, Suchindran CM (2012). Calcium intakes and femoral and lumbar bone density of elderly U.S men and women: National Health and Nutrition Examination Survey 2005-2006 analysis. J Clin Endocrinol Metab..

[22] Kelly PJ, Hopper JL, Macaskill GT, Pocock NA, Sambrook PN, Eisman JA (1991). Genetic factors in bone turnover. J Clin Endocrinol Metab..

[23] Pollitzer WS, Anderson JJ (1989). Ethnic and genetic differences in bone mass: a review with a hereditary vs environmental perspective. Am J Clin Nutr..

[24] Slovik DM, Adams JS, Neer RM, Holick MF, Potts JT Jr (1991). Deficient production of 1,25-dihydroxyvitamin D in elderly osteoporotic patients. N Engl J Med..

